# COVID-19 mortality, excess mortality, deaths per million and infection fatality ratio, Belgium, 9 March 2020 to 28 June 2020

**DOI:** 10.2807/1560-7917.ES.2022.27.7.2002060

**Published:** 2022-02-17

**Authors:** Geert Molenberghs, Christel Faes, Johan Verbeeck, Patrick Deboosere, Steven Abrams, Lander Willem, Jan Aerts, Heidi Theeten, Brecht Devleesschauwer, Natalia Bustos Sierra, Françoise Renard, Sereina Herzog, Patrick Lusyne, Johan Van der Heyden, Herman Van Oyen, Pierre Van Damme, Niel Hens

**Affiliations:** 1 Data Science Institute, I-BioStat, Universiteit Hasselt, Hasselt, Belgium; 2 I-BioStat, KU Leuven, Leuven, Belgium; 3 Interface Demography (ID), Department of Sociology, Vrije Universiteit Brussel, Brussels, Belgium; 4 Global Health Institute (GHI), Family Medicine and Population Health, University of Antwerp, Antwerp, Belgium; 5 Centre for Health Economics Research and Modelling of Infectious Diseases (CHERMID), Vaccine & Infectious Disease Institute (VAXINFECTIO), University of Antwerp, Antwerp, Belgium; 6 Centre for the Evaluation of Vaccination (CEV), Vaccine & Infectious Disease Institute (VAXINFECTIO), University of Antwerp, Antwerp, Belgium; 7 Department of Epidemiology and public health, Sciensano, Brussels, Belgium; 8 Department of Translational Physiology, Infectiology and Public Health, Ghent University, Ghent, Belgium; 9 Statistics Belgium, Brussels, Belgium; 10 Department of Public Health and Primary Care, Ghent University, Ghent, Belgium

**Keywords:** COVID-19 Mortality, Excess mortality, COVID-19 Deaths per Million, Infection Fatality Ratio

## Abstract

**Background:**

COVID-19 mortality, excess mortality, deaths per million population (DPM), infection fatality ratio (IFR) and case fatality ratio (CFR) are reported and compared for many countries globally. These measures may appear objective, however, they should be interpreted with caution.

**Aim:**

We examined reported COVID-19-related mortality in Belgium from 9 March 2020 to 28 June 2020, placing it against the background of excess mortality and compared the DPM and IFR between countries and within subgroups.

**Methods:**

The relation between COVID-19-related mortality and excess mortality was evaluated by comparing COVID-19 mortality and the difference between observed and weekly average predictions of all-cause mortality. DPM were evaluated using demographic data of the Belgian population. The number of infections was estimated by a stochastic compartmental model. The IFR was estimated using a delay distribution between infection and death.

**Results:**

In the study period, 9,621 COVID-19-related deaths were reported, which is close to the excess mortality estimated using weekly averages (8,985 deaths). This translates to 837 DPM and an IFR of 1.5% in the general population. Both DPM and IFR increase with age and are substantially larger in the nursing home population.

**Discussion:**

During the first pandemic wave, Belgium had no discrepancy between COVID-19-related mortality and excess mortality. In light of this close agreement, it is useful to consider the DPM and IFR, which are both age, sex, and nursing home population-dependent. Comparison of COVID-19 mortality between countries should rather be based on excess mortality than on COVID-19-related mortality.

## Introduction

Belgium’s coronavirus disease (COVID-19)-related mortality per million inhabitants has been reported as the highest worldwide (excluding microstates) between 11 April 2020 and 26 August 2020. For example, on 28 June 2020 Our World in Data [1] reported that Belgium had 830 COVID-19-related deaths per million population (DPM) vs 107 in Germany, 379 in the United States (US), 456 in France, 539 in Sweden, 574 in Italy and 593 in the United Kingdom (UK). Because of its relative nature, DPM appears to be an objective measure for comparison. However, it heavily depends on many factors, including but not limited to population density and the completeness of reporting on COVID-19 mortality [2]. During the first half year of 2020, the east coast of the US was primarily affected by COVID-19, resulting in a relatively low DPM for the entire US as compared to other countries. The high death toll observed on the east coast was diluted by the largely unaffected west coast population in the first half of the year. Indeed, in New York State until the end of June 2020, the DPM was 1,599, largely exceeding the Belgian DPM for this period [3]. The completeness of COVID-19-related mortality reporting itself also depends on many factors such as directives, availability of data and the definition of a COVID-19-related death. Belgium is one of the few countries whose COVID-19-related mortality notification criteria is broader than the WHO criteria [4] and includes laboratory and radiologically-confirmed COVID-19 deaths in hospital, nursing homes or other long-term care facilities as well as deaths in possible COVID-19 cases [5,6].

The case fatality ratio (CFR), another frequently reported measure regarding COVID-19-related mortality, is arguably also not a good basis for international comparison [7,8]. Besides its dependence on the accuracy of COVID-19-related mortality reporting, it is strongly influenced by testing strategies. Additionally, in some instances, the delay between case confirmation and death is not accounted for [9] and age dependency is ignored. The handling of suspected COVID-19 cases is ambiguous at best. However, the CFR can be useful as a tool to estimate global infection fatality ratio (IFR) [10], when the IFR is derived as a limit of the CFR by asymptotic models.

It is difficult to compare COVID-19 mortality in Belgium to countries that have a less extensive reporting strategy, in particular when the gap between excess mortality and COVID-19 mortality is large, such as in the Netherlands, Italy, or Austria [3]. Arguably, excess mortality is a better basis for international comparison [2,11].

To understand the subtleties of COVID-19 mortality in Belgium, we examined COVID-19-related mortality, placing it against the background of excess mortality in Belgium, and compared the COVID-19 DPM and IFR between countries and within subgroups in Belgium. Using the number of COVID-19 deaths, COVID-19 hospitalisations and seroprevalence estimates based on serial serological surveys [12], COVID-19 DPM and IFR were estimated overall and in relation to age and sex, and for the general population as a whole, the nursing home population (NHP) and the non-NHP, which excludes a small but very frail segment of the population, separately.

## Methods

The study period from week 11 to week 26 2020 was chosen to cover the first COVID-19 pandemic wave, for which accurate death counts are available following data cleaning. We do not consider the CFR, but will discuss the IFR.

### COVID-19 mortality

The Belgian institute for public health, Sciensano, registers daily COVID-19 deaths [13]. Daily mortality data were extracted on 30 September 2020 and were aggregated weekly in the age groups: 0–9, 10–19, 20–29, 30–39, 40–49, 50–59, 60–69, 70–79, 80–89 and ≥ 90 years. These 10 categories are used throughout the analyses, unless otherwise specified. The daily information was binned in Monday to Sunday weeks. Missing data redistribution methods [14] were used to redistribute deaths with missing age and/or sex in an ad hoc fashion over the corresponding week, so as to match the age-sex distribution observed from historical mortality data.

In addition, two sub-populations that jointly comprise the NHP deaths were considered: (i) nursing home residents who died in nursing homes and (ii) nursing home residents who died in hospitals. The latter information is registered by the hospitals in a separate dataset. Redistribution methods per week were used for deaths with missing information, matching the age-sex distribution observed from the nursing home residents’ mortality in hospitals in Belgium.

### COVID-19 case definition

Registered COVID-19-related deaths in Belgium include deaths of confirmed and possible COVID-19 cases. A case can be confirmed either by a chest computed tomography (CT) scan with clinical presentation or a laboratory test. Possible cases are those who meet the clinical criteria, whether or not there is an epidemiological link to a confirmed case [6,15].

### All-cause and excess mortality

Weekly mortality per sex and age category, for the years 2009–19 (complete) and for 2020 until 28 June, originated from the National Register. Statistics Belgium, the national statistical institute, processed these deaths and integrated them in Demobel, its demographic data warehouse [16]. Using the years 2009–19 combined, a weekly average profile termed baseline was obtained, with corresponding 99% pointwise prediction bands based on a normal distribution. The weekly average profiles were subtracted from the weekly mortality data of 2020 to estimate the weekly excess mortality, with corresponding 95% prediction intervals (PI).

### Population sizes and COVID-19 deaths per million

The sizes of the Belgian population (situation as at 1 January 2020) by age category and sex were taken from Statistics Belgium (Demobel), based on National Register data [17]. COVID-19 deaths per million were obtained by dividing COVID-19 mortality by the population sizes and adjusted by considering the ratio of the reported COVID-19-related mortality and excess mortality reported by Aron et al. [2]. Hence, the adjustment was based on excess mortality rather than COVID-19-related mortality and was called the excess DPM.

### Estimated number of SARS-CoV-2-infected individuals

We used a stochastic discrete-time age structured compartmental model [18] to estimate the number of SARS-CoV-2-infected individuals. For the purpose of a sensitivity analysis, the number of individuals infected with SARS-CoV-2 was additionally estimated with an individual-based model [19]. Both models were calibrated on national hospitalisation data and serial serological survey data [12]. The stochastic model additionally used Belgian mortality data [13] and the individual-based model employed doubling times [20]. Both models predicted the daily number of new infections per 10-year age groups. The individual-based model was developed to estimate SARS-CoV-2 infections in the general population and to measure the effect of the non-pharmaceutical interventions on the number of infections. It was not developed to estimate the number of infections per age category. Since the individual-based model assumed that older individuals live in relative isolation as compared to younger individuals, it is not well-suited to accommodate outbreaks in nursing homes, nor to reliably estimate the number of infections in the population aged over 80 years. Although the stochastic model did not explicitly account for elderly care homes, it did allow for substantial transmission in those higher age groups affected by outbreaks and transmission within nursing homes. Hence, the latter was deemed more reliable with regard to the estimation of the total number of SARS-CoV-2-infected individuals in the higher age categories.

### Infection fatality ratio

Inspired by the work of Nishiura et al. [21], the daily IFR was calculated as the number of deaths on day *t* who died because of SARS-CoV-2 infection and the total number of infections on day *t:*


$${IFR}_{t}=\frac{\sum_{j=0}^{K}{\hat{d}}_{t+j}f_{j\to t}}{i_{t}}$$

The predicted number of deaths, denoted by the symbol *d* is modelled according to a negative binomial regression, with the mean following a Richards model [22] at time *t* during the study period and beyond (*t* = 0, *. . ., T, . . . T* + *K*). The delay distribution between infection and death was estimated from the literature and from individual hospital survey data [23], with *ƒ _j→t_* representing the probability of a delay of *j* days between infection and death (*j* = 0, *. . ., K*) and *K* representing the maximal number of days between infection and death. The time between infection to symptom onset has a lognormal distribution with parameters 1.516 and 0.0164. The time between symptom onset and death was based on a Weibull distribution, which accounted for the interval-censoring nature of the observed delay times and truncation at the end of the study period and is age-specific [23]. Finally, the number of SARS-CoV-2-infected individuals *ἱ_t_* is the predicted mean number of SARS-CoV-2 infections based on the stochastic discrete time age-structured compartmental model [18] or the individual-based model [19].

The posterior distributions of the IFR at each time point were obtained by Markov Chain Monte Carlo (MCMC) sampling of the predicted number of deaths. A summary of the IFR between 11 March and 28 June 2020 was made by averaging the daily IFR. The 95% confidence interval (CI) of the IFR takes into account both the variability of the estimation of the SARS-CoV-2-infected individuals as well as the variability of the MCMC sampling. The IFR was estimated per age category, for the general population, the NHP and the non-NHP.

The data analysis was performed using SAS software version 9.4 (SAS Institute, Cary, US), GAUSS version 14 (Aptech Systems, Letchworth Garden City, UK), and R version 4.0.3 (R Foundation for Statistical Computing, Vienna, Austria); visualisations were made using Vega version 5.21.0 (https://vega.github.io/vega/).

### Ethical statement

No ethical approval was required for this study. There was no patient involvement and aggregated mortality data were used.

## Results

### COVID-19 mortality

Fewer than five COVID-19 deaths occurred in the combined age categories 0–29 years. In view of the low count, these age categories were excluded in the remainder of the analyses. Any measure based on these counts would be highly inaccurate. Of the COVID-19-related deaths in Belgium between week 11 to week 26 2020, 15 persons had neither age nor sex reported, 10 individuals had their age but not their sex reported (all aged 65 years or older), and one man was of unknown age. Given the low amount of missing data, the redistributed data do not influence the age or sex-related results.

**Figure 1:**
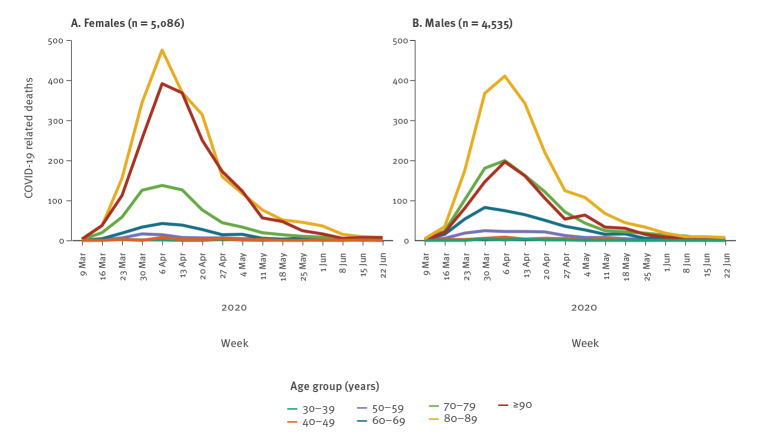
Age and sex specific COVID-19-related mortality, Belgium, 9 March–28 June 2020 (n = 9,621)

Of 9,621 COVID-19-related deaths, 4,535 were male and 5,086 were female (Supplementary Table S1). Among them, 2,591 (27%) were deaths of possible COVID-19 cases. The majority of the deaths in possible cases occurred in a nursing home (n = 2,310; 89%). The number of deaths strongly increases with age (Figure 1 and Supplementary Table S1). The peak of COVID-19 deaths was reached in week 15 (Figure 1 and Supplementary Table S1).

COVID-19: coronavirus disease.

With 4,763 deaths in nursing homes (15 individuals had missing age and sex in the reported data, while the sex of three individuals was not reported) and 1,294 nursing home residents who died in hospitals (129 with missing age and/or sex), the majority (63%) of COVID-19-related deaths occurred in the NHP (Supplementary Table S2 and S3). It is difficult to compare sexes in absolute terms, because the higher number of deaths in the female > 80 years-old age group, for example, is offset by the fact that the number of males in the > 80 years-old age group category is roughly half the number in the female category (Supplementary Table S4).

### Excess mortality

**Figure 2:**
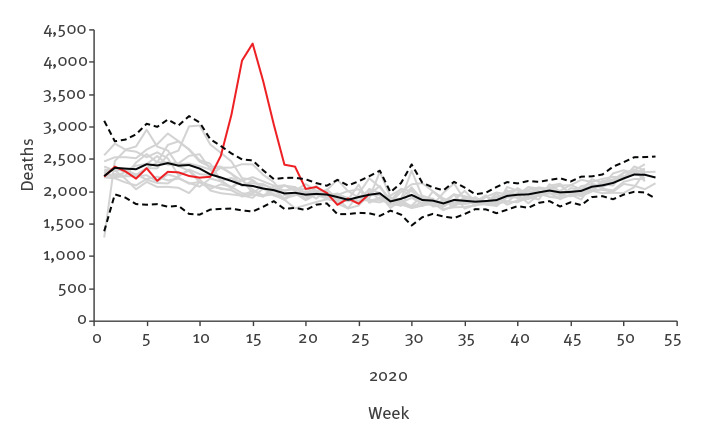
All-cause mortality, Belgium, 2009–2019 and weeks 1−26 2020

The excess mortality in 2020 is apparent when compared with the first 6 months of the years 2009–19 (Figure 2). The mortality until week 10 2020 was below the baseline (average over years 2009–19), although coherent with the prediction interval. It rose well over the seasonal variation of the historical mortality data in subsequent weeks. The mortality peak lies clearly outside the 99% pointwise prediction bands.

Grey curves refer to the period 2009–2019, with the black curve representing the average over 2009–2019 and dashed lines representing the 99% pointwise prediction bands. The red curve refers to 2020.

**Figure 3:**
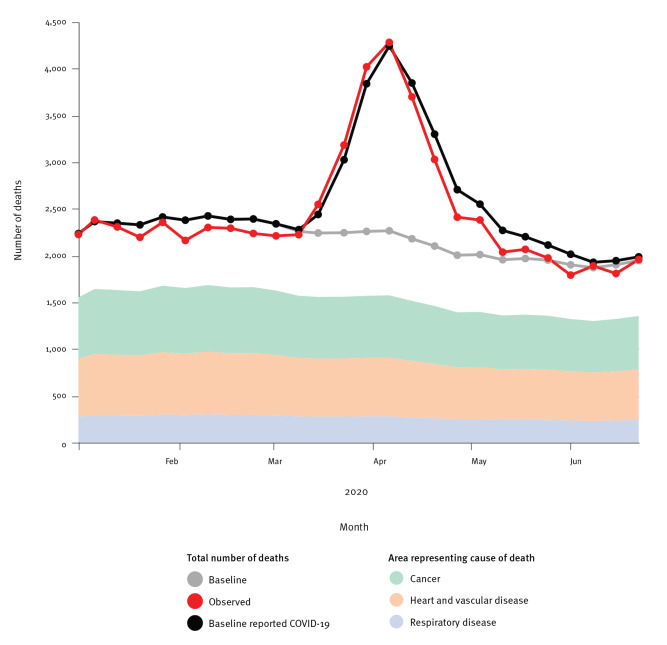
Observed all-cause mortality in 2020 vs average mortality (baseline) and COVID-19-related mortality combined and average mortality during 2009–2019 by cause of death, Belgium, weeks 1−26 2009–2020

The excess mortality in Belgium between weeks 11 and 26 2020, based on the weekly average from 2009 to 2019, is 8,985 (95% prediction interval: 5,388–12,582). There was a near coincidence of the excess all–cause and COVID-19 mortality (Figure 3) and the peak of excess mortality was strongly driven by the older age categories (Supplementary Figure S1).

COVID-19: coronavirus disease.

### COVID-19 deaths per million inhabitants

Over the study period, Belgium registered 837 DPM (Table 1). Similar to the reported COVID-19 deaths, a strong age and sex effect was observed in the DPM (Table 1). The DPM increased exponentially with increasing age, while in all age categories the DPM for males was higher than that for females. However, what was most striking is the NHP effect in terms of large absolute numbers of deaths. For the non-NHP, the overall figure declined to 438 DPM.

**Table 1:** COVID-19-related deaths per million inhabitants per age and sex group for the non-NHP, NHP and general population, Belgium, 9 March–28 June 2020

Characteristics		Age groups (years)						All ages combined	Over > 60 years of age combined
		25–49	50–59	60–69	70–79	80–89	≥ 90		
Female	Non-NHP	19	93	200	654	1,834	4,349	318	876
	NHP	NA		25,379	34,408	41,673	53,604	44,486	44,486
	General population	19	92	311	1,372	6,743	22,110	872	3,183
Male	Non-NHP	25	182	538	1,431	4,081	10,787	563	1,720
	NHP	NA		29,463	61,234	91,117	98,069	78,751	78,751
	General population	25	182	687	2,343	9,305	28,201	801	3,409
Both sexes									
Non-NHP		22	138	365	1,014	2,753	6,449	438	1,266
NHP		NA		27,391	44,633	53,495	61,464	53,267	53,267
General population		22	138	495	1,821	7,748	23,808	837	3,286

COVID-19: coronavirus disease; NA: not applicable; NHP: nursing home population.

When adjusting the DPM for the different degrees of reporting of COVID-19-related mortality by country, Belgium’s excess DPM of 755 is comparable to that of the UK (Table 2).

**Table 2:** Ranking of countries by COVID-19 deaths per million and excess deaths per million, 28 June 2020^a^

Country	COVID-19 deaths per million	Country/state/town	Excess deaths per million
Belgium	830	New York City, US	2,222
Spain	606	New York State, US	1,599
UK	593	Spain	1,010
Italy	574	Italy	857
Sweden	539	Belgium	755
France	456	UK	742
US	379	Netherlands	574
Netherlands	356	France	470
Germany	107	NA	

COVID-19: coronavirus disease; NA: not applicable; UK: United Kingdom; US: United States.

^a^ Data was obtained from Our World in Data [1].

### Infection fatality ratio

The number of SARS-CoV-2 infections in Belgium estimated by the stochastic model and the individual-based model were similar for the lower age categories (Supplementary Figure S2), while in the upper age categories they disagreed. This translated to similar IFR from both models when looking across all ages in the general population and the non-NHP (Supplementary Table S5). As expected for the higher age groups and in the NHP, the individual-based model overestimated the IFR.

**Figure 4:**
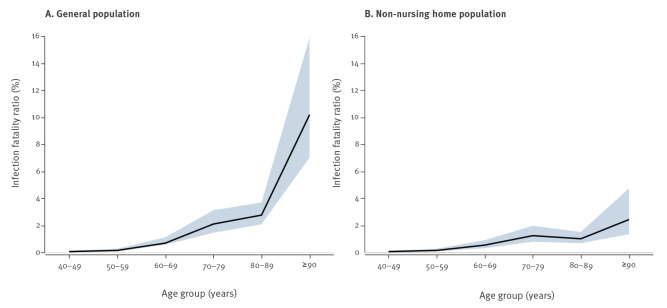
Infection fatality ratio in the general population (A) and the non-nursing home population (B), Belgium, 9 March–28 June 2020

Based on the stochastic model, the IFR across all ages was estimated to be 1.5% in the general population (Table 3). The IFR showed an age-dependent exponential increase, with nearly 0% under 40 years of age, increasing to 10% above 89 years of age in the general population (Figure 4).

**Table 3:** Infection fatality ratio for the non-nursing home population, nursing home population and the general population with the stochastic model, Belgium, 9 March–28 June 2020

Characteristics	Age groups (years)													
	40–49		50–59		60–69		70–79		80–89		≥ 90		All ages combined	
	IFR	95% CI	IFR	95% CI	IFR	95% CI	IFR	95% CI	IFR	95% CI	IFR	95% CI	IFR	95% CI
Non-NHP	0.05	0.01–0.13	0.14	0.07–0.26	0.53	0.30–0.90	1.23	0.78–1.96	1.00	0.67–1.50	2.42	1.34–4.73	0.58	0.42–0.81
NHP	NA				31.42	15.43–63.13	45.91	29.89–72.37	18.46	13.55–25.36	26.27	17.90–41.26	20.98	15.83–28.58
General population	0.05	0.01–0.13	0.14	0.07–0.26	0.68	0.56–1.11	2.09	1.44–3.11	2.75	2.07–3.69	10.18	7.01–15.90	1.47	1.14–1.94

CI: confidence interval; IFR: infection fatality ratio; NA: not applicable; NHP: nursing home population.

Black line refers to the infection fatality ratio and shaded area represents the 95% confidence interval.

The striking difference between COVID-19 mortality in the non-NHP and the NHP seen in the DPM was also evident in the IFR of 0.6% and 21% respectively. Depending on the age, the IFR in the NHP is 10 to 60-fold higher than in the non-NHP. Interestingly, when comparing the ratio of the IFR or DPM in the NHP vs the non-NHP within a particular age group, the ratio decreased with increasing age.

## Discussion

During the first wave of the pandemic, Belgium was often cited as being one of the worst hit countries worldwide with regards to COVID-19 mortality. Therefore, we studied in detail the COVID-19-related mortality, excess mortality, and its relation to COVID-19 DPM and IFR in Belgium in the first 6 months of 2020 in age- and nursing home-dependent subgroups, placing them in the perspective of internationally reported COVID-19-related mortality.

The COVID-19 mortality in Belgium underscores the severity of the epidemic. In the second week of April 2020, COVID-19 mortality was twice as high as the long-term average-mortality for that week, exceeding by far the influenza-related increases in mortality of the previous 10 years [24]. Belgium’s number of deaths in April 2020 was the highest among all months of April since World War II, although January 1951 and February 1960 saw similar figures [15,25].

The Belgian institute for public health, Sciensano, decided early on in the pandemic to report not only deaths from cases confirmed by COVID-19 laboratory tests or chest CT scans, but also deaths in possible COVID-19 cases [6]. Since COVID-19 mortality monitoring varies between countries, international comparisons may be seriously biased [11]. The close agreement between reported COVID-19-related mortality in Belgium (n = 9,621) and the excess mortality (n = 8,985) supports the reporting strategy in Belgium [15]. However, this coincidence is not a definitive proof that all excess deaths were COVID-19-related, although it has been reported internationally that around 90% of deaths suspected to be COVID-19-related are in fact COVID-19-related deaths [10]. While it may be possible, for example, that some excess deaths were related to other factors such as lockdown-induced stress, a plausible assumption can be made that such effects on mortality are minor [11]. Further examination is warranted as soon as the national cause-specific mortality database becomes available, typically after a 3-year interval.

The reported COVID-19-related deaths as a share of excess deaths is 107%, which is slightly different from the 110% reported by Aron et al. [2] because a thorough revision of the reported mortality related to cases of COVID-19 occurred after the publication of the paper and because we consider additional weeks and additional historical data. The difference between excess mortality and COVID-19-related deaths in Belgium might be because of the inaccuracy of predicting 2020 mortality with the weekly averaging method [11].

The mortality-related measures, DPM and IFR, are dependent on the testing strategy and the completeness of the mortality reporting. If one wants to compare COVID-19 mortality between countries, it would be better to take into account the possible under-reporting of mortality related to cases of COVID-19 and for example adjust the COVID-19 DPM by using estimates of the amount of under-reporting. Doing so, Belgium’s excess DPM is still high, but no longer an extreme.

In addition to the completeness of mortality reporting, the DPM and IFR depend on other important factors. Although DPM and IFR clearly increase with age, adjusting for age distribution of a country is of less importance when comparing European and western countries. Despite differences in the age distribution of populations between European countries, Canada and the US [26], most of these countries fluctuate at around 20% of the population above 65 years of age. The age distribution would be more important when comparing western vs African or Asian countries. A higher proportion of older people in a population may have various demographic reasons, such as low birth rate occurring during a few years, migration and an increasing level of general well-being. The impact of high-quality healthcare facilities on the demographic age distribution is more debatable, but with an increasingly aging population, underlying comorbidities such as high blood pressure and diabetes, which are known to be risk factors for COVID-19 mortality, are more prevalent. This may partly explain the increased mortality observed with age.

Directly related to this, but worth a separate mention, is the observation in both the DPM and IFR that the epidemic has been severe in the NHP in Belgium. The age-dependent decrease of the NHP vs the non-NHP COVID-19 DPM and IFR ratios suggests that a larger difference with respect to frailty and comorbidities exists between those subpopulations in the 60–79 years-old age group than in the > 80 years-old age group. It is indeed plausible to assume that if someone requires nursing and caring attention in a nursing home at age 60 years, they have some limiting comorbidities or increased frailty. In the NHP, the potential of caregivers as source of infection in addition to health status, should not be underestimated and protection and preventive measures should be taken in view of possible future outbreaks. In summary, the large DPM in the NHP vs the non-NHP, when compared within a given age, arguably results from a larger number of infections, in combination with an increased IFR. All in all, the epidemic’s impact on Belgian nursing homes was extremely serious, in line with international findings [27,28]. The IFR estimates in Belgium are similar to the ones reported for France by O’Driscoll et al. [29] of 0.7%, 22.3% and 1.1% in the non-NHP, NHP and general population, respectively. Compared with the meta-analysis of Levin et al. [30], we find a lower IFR in the population aged 70 years and older.

In European and western countries, many other factors influence the COVID-19-related DPM and IFR including: (i) international connectivity and internal contact patterns; (ii) the population density in a country, which depends among other factors on the size and geographical dispersion of a country; (iii) the timing of the epidemic i.e. the mortality should be compared relative to a well-defined baseline e.g. 50 days since the first day at which the DPM exceeded 1.0, rather than calendar time. This would produce, for example: Italy, 24 April, 423; France, 7 May, 443; UK, 7 May, 443; Belgium, 8 May, 726; Sweden, 10 May, 319; US, 11 May, 240; and Germany, 12 May, 90; (iv) the varying measures taken by national and regional authorities to fight the epidemic; (v) differences in healthcare systems and; (vi) socioeconomic status.

Our study has several limitations. Although the IFR is a useful measure to compare COVID-19 mortality, the number of SARS-CoV-2 infections is an additional source for bias and uncertainty. This supports the use of sensitivity analysis by applying different methods to estimate the number of SARS-CoV-2 infected individuals, along with the reporting of interval estimates. The methods used to estimate the number of SARS-CoV-2-infected individuals in our analysis could potentially have been improved by the use of seroprevalence data specific to the NHP; however, given the severity of the epidemic, collection thereof was not straightforward. These data are largely unavailable at the moment. The compartmental and individual-based models used to estimate the number of SARS-CoV-2 infections now assume a similar seroprevalence in the NHP as the non-NHP.

## Conclusion

During the first wave of the COVID-19 pandemic, Belgium had virtually no discrepancy between COVID-19-related mortality and excess mortality, supporting its mortality reporting strategy. In light of the close agreement, it is useful to consider the Belgian COVID-19 DPM and IFR, which are both age, sex and NHP-dependent. The steep age-related gradient in mortality contributes useful information to policymakers for differential non-pharmaceutical interventions. Comparison of COVID-19 mortality between countries should be based on excess mortality rather than reported mortality. However, a more detailed study and further international comparison of COVID-19 mortality is needed for the ongoing pandemic, also in view of the non-pharmaceutical interventions implemented, antiviral medication, as well as vaccine uptake and effectiveness.

## Note

The COVID-19 mortality data, collected by Sciensano, are available in the open data mortality repository, https://epistat.wiv-isp.be/covid/. For additional data, a data request form (https://epistat.wiv-isp.be/datarequest/index.aspx) has to be returned to the Data Protection Officer office at Sciensano..

## Supplementary Data

253,000 Supplement
