# Supplementary material for: COVID-19 mortality, excess mortality, deaths per million and infection fatality ratio, Belgium, 9 March 2020 to 28 June 2020
Source: Euro Surveill. 2022 Feb 17;27(7):2002060. doi: 10.2807/1560-7917.ES.2022.27.7.2002060 (PMC8855510; doi:10.2807/1560-7917.ES.2022.27.7.2002060)
Supplement: Supplement [file 20-02060_MOLENBERGHS_Supplement.pdf]

Supplementary material

This supplementary material is hosted by Eurosurveillance as supporting information alongside the article “COVID-19 mortality, excess mortality, deaths per million and infection fatality ratio, Belgium, 9 March 2020 to 28 June 2020” on behalf of the authors who remain responsible for the accuracy and appropriateness of the content. The same standards for ethics, copyright, attributions and permissions as for the article apply. Supplements are not edited by Eurosurveillance and the journal is not responsible for the maintenance of any links or email addresses provided therein.

Table S1: COVID-19 mortality in the general population in Belgium per age category, sex and week

	Week																
Age	11	12	13	14	15	16	17	18	19	20	21	22	23	24	25	26	Total
Male																	
30–39	0	0	0	<5	<5	<5	<5	<5	0	0	0	0	0	0	0	0	9
40–49	0	<5	<5	5	8	<5	5	<5	0	5	0	0	<5	0	0	0	35
50–59	0	5	18	24	22	22	21	12	7	7	<5	<5	0	<5	<5	0	146
60–69	<5	15	53	82	74	64	50	35	26	15	16	5	8	<5	<5	<5	453
70–79	<5	23	101	180	199	162	121	71	43	24	21	18	13	11	<5	6	1001
80–89	<5	34	174	367	410	342	219	124	107	67	44	33	18	9	9	7	1968
90+	<5	20	80	146	196	161	105	53	63	33	30	15	8	5	<5	<5	923
Total	11	99	428	807	910	757	522	300	246	151	115	73	48	30	19	19	4535
Female																	
30–39	0	<5	<5	<5	<5	0	0	<5	<5	0	<5	<5	0	0	<5	0	12
40–49	0	0	<5	0	7	<5	<5	<5	<5	<5	0	0	<5	0	<5	0	22
50–59	0	<5	6	16	14	7	6	6	<5	5	<5	<5	<5	<5	0	0	73
60–69	0	<5	18	33	42	38	27	14	15	5	<5	5	<5	<5	<5	<5	214
70–79	<5	19	58	125	137	126	76	44	33	19	14	10	8	5	<5	<5	682
80–89	<5	36	155	344	475	369	314	159	117	76	51	45	36	15	9	7	2211
90+	<5	37	112	254	391	368	250	172	123	56	47	24	16	5	7	7	1872
Total	8	99	353	773	1067	910	675	401	294	163	118	88	65	28	24	20	5086
Male+Female																	
30–39	0	<5	<5	<5	<5	<5	<5	<5	<5	0	<5	<5	0	0	<5	0	21
40–49	0	<5	<5	5	15	5	7	8	<5	7	0	0	<5	0	<5	0	57
50–59	0	6	24	40	36	29	27	18	11	12	6	5	<5	<5	<5	0	219
60–69	<5	19	71	115	116	102	77	49	41	20	19	10	10	6	6	<5	667
70–79	6	42	159	305	336	288	197	115	76	43	35	28	21	16	6	10	1683
80–89	7	70	329	711	885	711	533	283	224	143	95	78	54	24	18	14	4179
90+	<5	57	192	400	587	529	355	225	186	89	77	39	24	10	10	11	2795
Total	19	198	781	1580	1977	1667	1197	701	540	314	233	161	113	58	43	39	9621

Table S2: COVID-19 mortality in the NHP in Belgium per age category, sex and week

Age	Week 11	12	13	14	15	16	17	18	19	20	21	22	23	24	25	26	Total
**Male**																	
60–69	0	0	10	16	30	17	10	<5	5	<5	<5	<5	<5	<5	0	0	101
70–79	0	5	26	73	100	71	48	28	18	11	6	<5	5	<5	<5	0	399
80–89	<5	10	55	191	295	221	138	74	68	36	30	18	7	<5	<5	<5	1157
90+	0	10	36	98	138	126	77	44	53	23	15	10	<5	<5	<5	<5	640
Total	<5	26	128	379	563	434	273	148	144	72	54	31	19	10	7	5	2297
**Female**																	
60–69	0	<5	<5	12	18	20	8	<5	6	<5	<5	0	<5	0	<5	0	77
70–79	0	<5	16	56	90	79	46	23	22	7	9	<5	<5	<5	<5	<5	364
80–89	<5	24	76	247	393	303	255	126	89	59	41	28	23	8	7	<5	1684
90+	0	28	75	214	357	319	227	164	104	57	38	22	15	<5	7	6	1636
Total	<5	56	169	530	858	722	536	316	221	125	89	53	44	14	17	11	3761
**Male+Female**																	
60–69	0	<5	12	29	48	37	18	5	11	<5	<5	<5	5	<5	<5	0	177
70–79	0	9	42	130	190	150	94	51	40	18	15	5	9	6	<5	<5	763
80–89	<5	34	131	438	688	524	394	200	157	95	71	46	30	12	11	5	2841
90+	0	38	111	312	495	445	304	208	157	80	53	32	19	5	9	9	2277
Total	<5	82	296	909	1420	1156	809	464	365	197	143	84	63	24	24	16	6057

Table S3: COVID-19 mortality in the non-NHP in Belgium per age category, sex and week

	Week																
Age	11	12	13	14	15	16	17	18	19	20	21	22	23	24	25	26	Total
Male																	
30–39	0	0	0	<5	<5	<5	<5	<5	0	0	0	0	0	0	0	0	9
40–49	0	<5	<5	5	8	<5	5	<5	0	5	0	0	<5	0	0	0	35
50–59	0	5	18	24	22	22	21	12	7	7	<5	<5	0	<5	<5	0	146
60–69	<5	15	43	66	44	47	41	33	21	13	13	<5	5	<5	<5	<5	352
70–79	<5	18	75	107	99	91	73	43	25	13	15	16	8	7	<5	6	602
80–89	<5	24	119	176	115	121	81	50	39	31	14	15	11	5	5	5	811
90+	<5	10	44	48	58	35	28	9	10	10	15	5	<5	<5	<5	<5	283
Total	8	73	300	428	347	323	249	152	102	79	61	42	29	20	12	14	2238
Female																	
30–39	0	<5	<5	<5	<5	0	0	<5	<5	0	<5	<5	0	0	<5	0	12
40–49	0	0	<5	0	7	<5	<5	<5	<5	<5	0	0	<5	0	<5	0	22
50–59	0	<5	6	16	14	7	6	6	<5	5	<5	<5	<5	<5	0	0	73
60–69	0	<5	16	21	24	18	19	11	9	<5	<5	5	0	<5	<5	<5	137
70–79	<5	16	42	69	47	47	30	21	11	12	5	7	<5	<5	<5	<5	318
80–89	<5	12	79	97	82	66	59	33	28	17	10	17	13	7	<5	<5	527
90+	<5	9	37	40	34	49	23	8	19	<5	9	<5	<5	<5	0	<5	236
Total	7	43	185	243	209	188	139	85	73	38	29	35	21	14	7	9	1325
Male+Female																	
30–39	0	<5	<5	<5	<5	<5	<5	<5	<5	0	<5	<5	0	0	<5	0	21
40–49	0	<5	<5	5	15	5	7	8	<5	7	0	0	<5	0	<5	0	57
50–59	0	6	24	40	36	29	27	18	11	12	6	5	<5	<5	<5	0	219
60–69	<5	18	59	86	68	65	60	44	30	16	15	9	5	5	<5	<5	490
70–79	6	33	117	175	146	138	103	64	36	25	20	23	12	10	<5	8	920
80–89	<5	36	198	273	197	187	139	83	67	48	24	32	24	12	7	9	1339
90+	<5	19	81	88	92	84	51	17	29	9	24	7	5	5	<5	<5	518
Total	15	116	485	671	557	511	388	237	175	117	90	77	50	34	19	23	3564

Table S4: Population of Belgium per age category and sex

Sex	Age group 0–24	25–49	50–59	60–69	70–79	80–89	90+	Total
				non-NHP				
Female	1,584,423	1,861,034	789,284	685,111	486,553	287,481	54,148	5,746,561
Male	1,653,075	1,875,264	801,344	655,566	420,643	198,801	26,203	5,628,845
Total	3,237,498	3,736,298	1,590,628	1,340,677	907,196	486,282	80,351	11,375,406
				NHP				
Female	NA	NA	NA	3,034	10,579	40,410	30,520	86,016
Male	NA	NA	NA	3,428	6,516	12,698	6,526	31,219
Total	NA	NA	NA	6,462	17,095	53,108	37,046	117,235
				General population				
Female	1,584,423	1,861,034	789,284	688,145	497,132	327,891	84,668	5,832,577
Male	1,653,075	1,875,264	801,344	658,994	427,159	211,499	32,729	5,660,064
Total	3,237,498	3,736,298	1,590,628	1,347,139	924,291	539,390	117,397	11,492,641

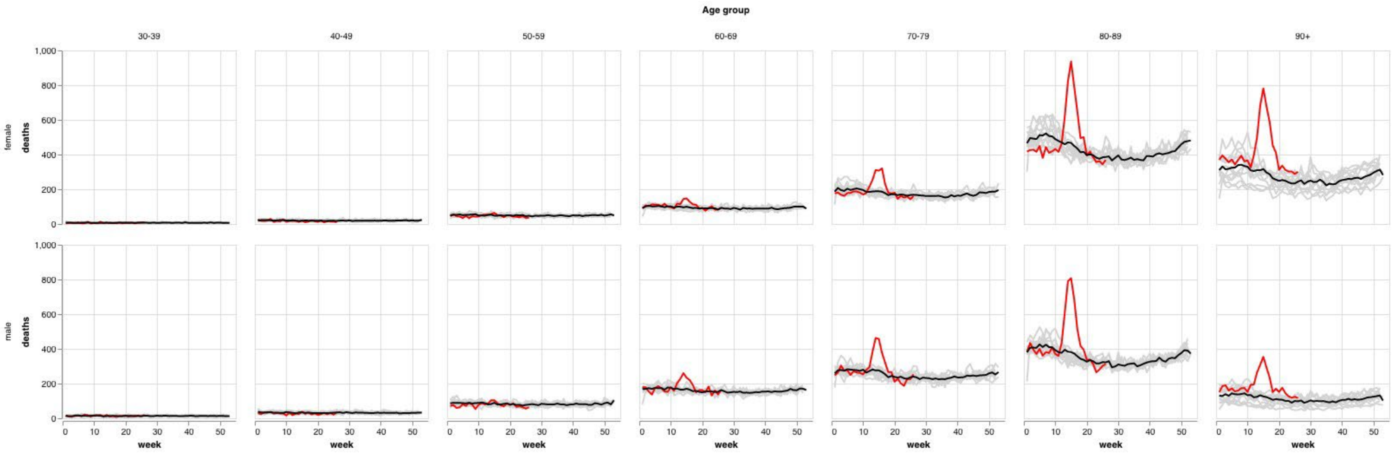

Figure S1: All-cause mortality by age group and gender in Belgium. Grey curves refer to years 2009– 2019, with the black curve the average over 2009– 2019. The red curve refers to year 2020.

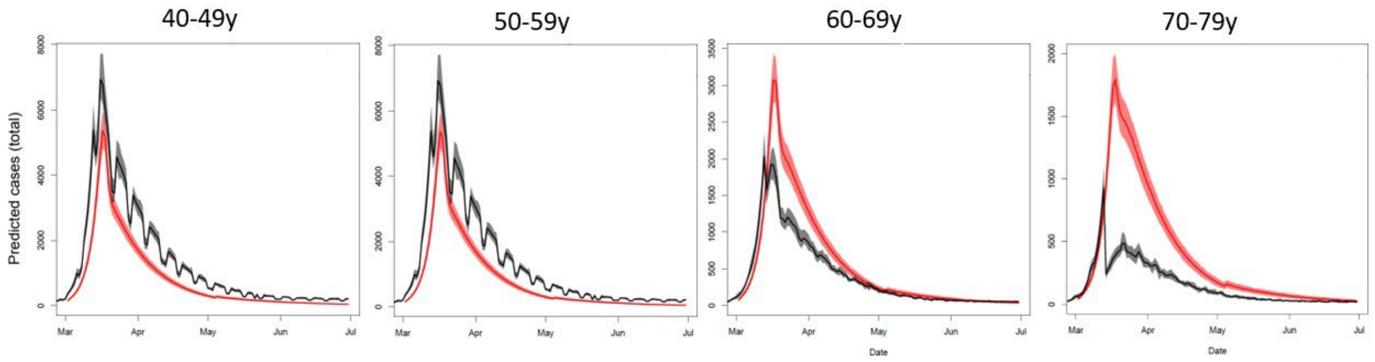

Figure S2: Estimated number of SARS-CoV-2 infections by age group in Belgium. Red curves refer to the estimation by the stochastic compartmental model and black curves to the estimation by the individual-based model.

Table S5: Infection fatality ratio in Belgium with 95% confidence interval per age category for the non-NHP and the general population with the individual-based model.

	Age				
	40–49	50–59	60–69	70–79	All Ages combined
non-NHP	0.03 (0.01–0.06)	0.12 (0.06–0.20)	0.61 (0.38–0.95)	2.16 (1.42–3.30)	0.65 (0.50-0.84)
General population	0.03 (0.01–0.06)	0.12 (0.06–0.20)	0.79 (0.52–1.18)	3.72 (2.64–5.35)	1.72 (1.39–2.12)
